# Fitness-Related Activities and Medical Claims Related to Hospital Admissions — South Africa, 2006

**Published:** 2009-09-15

**Authors:** Estelle V. Lambert, Rosanne da Silva, Libero Fatti, Deepak Patel, Tracy Kolbe-Alexander, Wayne Derman, Adam Noach, Craig Nossel, Thomas Gaziano

**Affiliations:** UCT/MRC Research Unit for Exercise Science and Sports Medicine, University of Cape Town; University of the Witwatersrand, Johannesburg, South Africa; University of the Witwatersrand, Johannesburg, South Africa; University of Cape Town, Cape Town, South Africa, and Discovery Health, Johannesburg, South Africa; University of Cape Town, Cape Town, South Africa; University of Cape Town, Cape Town, South Africa; Discovery Health, Johannesburg, South Africa; Discovery Health, Johannesburg, South Africa; Brigham and Women’s Hospital, Harvard Medical School, Boston, Massachusetts

## Abstract

**Introduction:**

We report on the effect of an incentive-based wellness program on medical claims and hospital admissions among members of a major health insurer. The focus of this investigation was specifically on fitness-related activities in this insured population.

**Methods:**

Adult members of South Africa's largest private health insurer (n = 948,974) were grouped, a priori, on the basis of documented participation in fitness-related activities, including gym visits, into inactive (80%, equivalent to ≤3 gym visits/y), low active (7.0%, 4-23 gym visits/y), moderate active (5.2%, 24-48 gym visits/y), and high active (7.4%, >48 gym visits/y) groups. We compared medical claims data related to hospital admissions between groups after adjustment for age, sex, medical plan, and chronic illness benefits.

**Results:**

Hospitalization costs per member were lower in each activity group compared with the inactive group. This same pattern was demonstrated for admissions rates. There was good agreement between level of participation in fitness-related activities and in other wellness program offerings; 90% of people only nominally engaged in the wellness program also were low active or inactive, whereas 84% of those in the high active group also had the highest overall participation in the wellness program.

**Conclusion:**

Participation in fitness-related activities within an incentive-based health insurance wellness program was associated with lower health care costs. However, involvement in fitness-related activities was generally low, and further research is required to identify and address barriers to participation in such programs.

## Introduction

Physical activity can reduce illnesses and deaths linked to chronic diseases (1,2). The health benefits of physical activity increase with increasing frequency, duration, and intensity of exercise (2-4). Data from longitudinal cohort studies suggest that physical inactivity is associated with at least a 1.5-fold to 2.0-fold higher risk of most chronic diseases of lifestyle, such as coronary heart disease, type 2 diabetes, and hypertension (1,5), and accounts for an estimated 1.3% of lost disability-adjusted life-years worldwide. Furthermore, studies corroborate the public health recommendation that 30 minutes of accumulated, moderate-to-vigorous intensity physical activity on most days is protective for these chronic diseases (3). The associated risk of inactivity is similar in magnitude to many other well-known risk factors, such as overweight, smoking, hyperlipidemia, and low fruit and vegetable intake (1,6).

Cross-sectional studies have estimated the economic costs associated with inactivity, or the cost savings associated with regular physical activity, at a national level in many industrialized countries (7-9). In Canada, where more than two-thirds of the population is considered to be insufficiently active, physical inactivity is estimated to be responsible for 2.5% of the total direct health care costs or the equivalent of 21,000 lives lost prematurely each year (7). Efforts to model the cost of inactivity to health care plans have typically yielded similar or higher costs compared with national estimates. Using a cost-of-illness approach, another study examined medical claims among approximately 1.5 million health plan members aged 18 years or older in Minnesota (10). In this model, more than 30% of cases of stroke, cancer of the colon, cardiovascular disease, and osteoporosis were attributable to inactivity. Health care providers are recognizing the role of physical activity in reducing risk for noncommunicable diseases (11).

A small number of studies have demonstrated actual reduction in health care costs and cost savings in physically active members of health plans (12-14). For example, claims data from approximately 23,000 health plan members showed that average annual health care claims were approximately $250 lower for those who were either moderately active (1-2 times per week) or very active (3-4 times per week), compared with their sedentary counterparts, on the basis of self-report (12). After 2 years, Medicare members who received a health club benefit as part of their health plan had significantly fewer inpatient admissions and lower total health care costs than did matched controls (13).

Further evaluation of physical activity programs offered by health plans is needed to establish the cost savings of such strategies. We designed our study to examine the association between levels of participation in fitness-related activities, as part of the incentive-based wellness program Vitality on medical claims and hospital admissions among members of the largest national private health insurer in South Africa, Discovery Health.

## Methods

### Data source

In South Africa, participation in private medical plans is inversely associated with income, despite the copayment by employers. Only 34% of people earning above R5,000 (US $600) per month are members of private medical plans, and this proportion more than doubles at incomes of R10,000 (US $1,200) or more. Discovery Health is more than 3 times the size of its nearest competitor and alone accounts for approximately 35% of the open plan market and 25% of all medical plan beneficiaries in South Africa.

Discovery Health has offered Vitality*,* an incentive and reward-based health promotion program, to its members since 1998. Membership is voluntary and offered separately from the health plan because legislation in South Africa precludes differential insurance premiums based on health status or engagement with health promotion programs. The program is offered to plan members for a nominal monthly fee of approximately R100 (US $12) per family. The sample included both principal and spouse members whose benefits had been effective for a full 12 months during 2006 and who, during that time, were either registered for Vitality or were not registered for at least a full 12 months.

The final sample was 948,974 members. All data were analyzed unlinked to any personal identifiers. The Research Ethics Committee of the Faculty of Health Sciences, University of Cape Town, approved the study protocol.

### Levels of engagement in Vitality

The activities of the program are fitness-related activity, assessment and screening, healthy choices, and health knowledge. Specific activities include subsidized gym memberships, visits to dietitians and exercise specialists, smoking cessation and weight reduction programs, and access to online or in-person risk assessments and online and print media material for health and wellness. Participation in the various wellness services and programs earns the participants points, which we used as a proxy measure for the level of participation in the health promotion program. Points are redeemable as discounts (ranging from 15% to 45%) on various goods and services. The level of engagement in Vitality was classified as 1) not registered for Vitality; 2) registered for Vitality but with no points in any of the 4 categories, defined as registered but not engaged; 3) registered for Vitality and accumulating up to a threshold level of points, defined as low engagement; and 4) registered for Vitality and accumulating more than the threshold level of points, defined as high engagement.

### Categories of fitness-related activity

Vitality program participants were awarded points specifically for fitness-related activities according to the total number of recorded gym visits to participating commercial fitness center partners. Members could also accumulate points for participation in major sporting or fitness events such as road running or cycle races (members register to participate through a commercial partner organization, SA Active). The fitness status was defined as 1) high active — points equivalent to more than 48 gym visits per year; 2) moderate active — points equivalent to 24 to 48 gym visits per year; 3) low active — points equivalent to 4 to 23 gym visits per year; and 4) inactive — points equivalent to 3 or fewer gym visits per year.

### Claims data categories

Members of the Discovery Health insurance plan can subscribe to 2 different plan types: comprehensive and core. Plans differ in the degree of coverage for ambulatory care. Coverage does not substantially differ for conditions requiring hospital admission. Members with specified chronic conditions, such as hypertension, diabetes, and hypercholesterolemia, were expected to register for chronic illness benefits paid from the insurer's risk pool (as opposed to the member's personal medical savings account).

For this analysis hospital claims data included the admission rate, cost per member for the entire population, cost per patient admitted to a hospital, number of days hospitalized per patient, number of hospitalizations per patient, length of stay per patient, and cost per hospitalization. Because the insurance pool does not cover claims for acute ambulatory care, we considered those data as incomplete; therefore, they were not analyzed. A subsample analysis included only those members who had been hospitalized at least once.

A further diagnosis-related subgroup analysis was conducted for high active status compared with all other fitness-related activity groups for hospital admission rates. Preselected subgroups included cancers, cardiovascular diseases, musculoskeletal conditions, and endocrine and metabolic conditions such as diabetes, which are complications of conditions responsive to interventions for health risk behaviors.

### Statistical analysis

The adjusted means were first calculated for those engaged in the Vitality program, taking into account the effect of the weighted covariates. Factors that were likely to independently influence medical claims data, irrespective of participation in the wellness program, were preselected as covariates for the multivariate analysis of covariance (ANCOVA), with the Tukey-Kramer *t* test (for multiple comparisons) to determine significance. This approach combines regression with experimental design into a single model (15,16).

The covariates selected included age (in 5-year bands), sex, chronic illness status (single or multiple risk factors or comorbid conditions), and health plan options. We used a tree analysis implemented in SAS Enterprise Miner (SAS Institute, Inc, Cary, North Carolina) to assess the effect of these covariates under each claim cost category.

## Results

In 2006, approximately 60% of all members were registered for Vitality. Of these, 71% were inactive and 12% were high active (Table 1).

Men represented 59% of the high active group and 46% to 50% of all other groups. The proportion of people registered for chronic illness benefits was higher among people not registered for Vitality than people in any of the other groups.

### Fitness-related activity group and Vitality engagement

A strong relationship existed between engagement in fitness activities and engagement in Vitality (Table 2). Approximately 84% of the high active group had a high level of engagement with the Vitality program. Approximately 27% of those highly engaged in Vitality went to gym more than 96 times in 2006 (average of 1.9 times per week), and approximately 62% reported going to the gym more than 48 times. We did not determine the level of participation in other wellness activities. Those who were highly engaged in the Vitality program also had proportionally more participation in health knowledge activities (eg, online health risk assessment and feedback, online nutrition assessment and feedback) (56% vs 17%) and assessments and screening (48% vs 8%) compared with those who had low engagement.

### Fitness-related activity group and hospitalization

For members who were admitted to a hospital in 2006, the adjusted means for cost per patient, total number of days hospitalized per patient, number of admissions per patient, length of stay per patient, and cost per hospitalization were significantly lower in the high active group compared with all other groups (*P* < .001) (Table 3). In addition, number of days hospitalized per patient and length of stay were significantly lower among moderate active patients compared with those not registered or who were low active (*P* < .001). Among those patients with at least 1 hospital admission, both costs per patient and number of days of hospitalization per patient decreased in relation to increased levels of participation in fitness-related activities. The high active members who were hospitalized in 2006 experienced a mean annual savings in associated medical claims of R5,025 (US $603) compared with inactive members.

The cost per member, number of admissions per member, and length of stay per member were all significantly lower in the high active group (Table 4). Furthermore, hospitalization costs per member decreased in each group from the inactive to the high active group (*P* < .001). This same pattern was demonstrated for admissions rate (*P* < .001). Participants in the high active group saved an average of R1,535 (US $184) in health care costs compared with the inactive group.

### Diagnosis-related subgroup analysis

The admission rates per member for the high active group were significantly lower when compared with all other groups for diagnosis-related subgroups (Table 5). Admissions associated with cancer and mental illness were approximately 35% lower, and admissions associated with endocrine, nutritional, and metabolic disorders and kidney and urinary tract disorders were 20% lower.

## Discussion

Our study found an unequivocal and inverse relationship between fitness-related activities among insured persons and hospital claims and admissions. The cost savings were similar to those reported in previous research (12,13); average annual health care costs were approximately $250 lower among active compared with inactive members, even considering those who exercised only 1 to 2 times per week. A study of Medicare members receiving a health club membership as part of their health plan had fewer inpatient admissions and lower total health care costs than matched controls not receiving the benefit (13). The actual uptake of this benefit remained low; less than 7% of the total plan membership participated.

Differences in savings between studies may be explained, in part, by the fact that we analyzed only medical claims associated with hospitalization, whereas the comparable studies typically report total health care expenditures. Furthermore, study populations differed in terms of age and demographics. Among health plan members aged 50 years or older, changing physical activity status from inactive to active was associated with approximately $2,000 in savings in health care claims during 2 years compared with remaining inactive during the same time (14).

The strength of our study is that gym visits and sports participation were documented and not based on self-report. However, it may be argued that the definition of engagement in fitness-related activities (eg, >48 gym visits per year) lacked sufficient sensitivity to accurately reflect dose-response exposure to physical activity. These criteria are not compatible with physical activity and public health recommendations of 30 minutes of moderate-to-vigorous intensity activity on most, preferably all, days of the week (17). Despite this, the apparent dose-response effect suggests that the definitions may be sufficiently discriminating. Previous cohort studies have found that even 1 to 2 bouts of physical activity per week showed significant risk reduction for diabetes mellitus (18,19) and cardiovascular mortality (20).

The facts that the activity participation was documented and that most of the high active people were highly engaged in Vitality suggest that the potential health benefits that accrued were in part related to participation, even if there was potential for selection bias. Furthermore, the significant association between participation in fitness-related activities and reduced medical claims or admissions was also demonstrated in the disease-related subgroups. This is in line with cohort studies in which relative risk for cardiovascular death, for example, in women diagnosed with diabetes decreased by 7% with as little as 1 to 2 hours of moderate physical activity per week (21). Similarly, as much as a 40% savings in health care expenditures was demonstrated in members of a managed care cohort with diabetes who attended a community-based fitness program at least once per week (22).

We cannot rule out the fact that physical activity clusters with other positive health behaviors. This underlying association may explain, at least in part, some of the relationship between gym visits and medical claims. For example, in the Aerobics Center Longitudinal Study cohort, cardiorespiratory fitness was inversely associated with consumption of dietary fat, saturated fat, and cholesterol in approximately 10,000 people measured during an 8-year period (23).

The challenge remains that while those members who are highly engaged have significantly lower health care claims and hospitalization, they are underrepresented in the larger plan membership. Various health care providers have developed strategies to increase adoption of physically active lifestyles, including full or partial subsidy of a health club or fitness center memberships. Third-party monitoring of fitness center visits was associated with increased use (24). We cannot say whether the incentives and rewards associated with Vitality influenced participation in fitness-related activities.

The data concerning fitness-related activities were limited in that not all members would have submitted information or recorded gym visits. Alternatively, members may have participated in physical activities that the rewards program did not capture. This is the case particularly for members not registered in the Vitality program.

Participation in fitness-related activities within an incentive-based health insurance wellness program was associated with significantly lower health care costs. However, as in other studies, the involvement in fitness-related activities was generally low, and further research is required to identify and address the barriers to participation in such programs.

## Figures and Tables

**Table 1:** Demographic and Medical Plan Characteristics of Medical Plan Members (N = 948,974) by Fitness Activity Status, Discovery Health, South Africa, 2006

Category	Fitness Activity Status^a^				

	Not Registered for Vitality	Inactive	Low Active	Moderate Active	High Active
Members, n (%)	357,840 (37.7)	419,187 (44.2)	52,713 (5.6)	49,633 (5.2)	69,601 (7.3)
Vitality members in each fitness-related category, %	NA	70.9	8.9	8.4	11.8
Mean age, y	50.5	42.5	37.5	39.3	41.1
Men, %	45.5	48.0	48.1	49.8	58.9
Members registered for chronic conditions,^b^ %	28.9	15.4	10.4	12.5	13.1
Members on Comprehensive plan,^c^ %	40.9	50.3	49.0	48.5	49.1

Abbreviation: NA, not applicable.

a Fitness activity status definitions: 1) not registered — not registered in the Vitality health promotion program; 2) inactive — earned points equivalent to 3 or fewer gym visits per year; 3) low active — earned points equivalent to 4 to 23 gym visits per year; 4) moderate active — earned points equivalent to between 24 to 48 gym visits per year; and 5) high active — earned points equivalent to more than 48 gym visits per year.

b Members with specified chronic conditions such as hypertension, diabetes, and hypercholesterolemia were expected to register for chronic illness benefits paid from the insurer's risk pool (as opposed to the member's personal medical savings account).

c Discovery Health offers 2 plan types: comprehensive and core. Plans differ in the degree of coverage for ambulatory care, but coverage for conditions requiring hospital admission does not substantially differ.

**Table 2:** Agreement Between Engagement in the Vitality Wellness Program and Fitness Activity Status, Discovery Health, South Africa, 2006

Vitality Engagement Status^b^	Fitness Activity Status^a^, %				

	Inactive	Low Active	Moderate Active	High Active	Total^c^
Not registered	47	0	0	0	38
Registered but not engaged	27	0	0	0	22
Low engagement	24	90	72	16	31
High engagement	1	9	28	84	10

a Fitness activity status definitions: 1) not registered — not registered in the Vitality health promotion program; 2) inactive — earned points equivalent to 3 or fewer gym visits per year; 3) low active — earned points equivalent to 4 to 23 gym visits per year; 4) moderate active — earned points equivalent to between 24 to 48 gym visits per year; and 5) high active — earned points equivalent to more than 48 gym visits per year.

b Vitality engagement levels are 1) not registered for Vitality; 2) registered for Vitality but did not earn points for physical activity, defined as registered but not engaged; 3) registered for Vitality and accumulating points for physical activity, defined as low engagement; and 4) registered for Vitality and accumulating points for physical activity above the threshold, defined as high engagement.

c Totals do not equal 100 because of rounding.

**Table 3:** Adjusted Means Per Member for Hospitalized Members, by Fitness Activity Status, Discovery Health, South Africa, 2006

**Fitness Activity Status** a	Cost per Patient, R^b^ (95% CI)^c^	Length of Hospitalization per Patient, d (95% CI)^c^	No. of Admissions per Patient (95% CI)^c^	Cost per Hospitalization, R (95% CI)^c^	Length of Stay, d (95% CI)^c^
Not registered	30,455 (30,054-30,856)	6.1 (6.1-6.2)	1.6 (1.6-1.7)	18,497 (18,927-19,402)	3.6 (3.6-3.6)
Inactive	31,373 (30,924-31,822)	5.9 (5.8-6.0)	1.6 (1.6-1.6)	19,164 (18,927-19,402)	3.4 (3.4-3.5)
Low active	30,112 (29,168-31,057)	5.4 (5.2-5.5)	1.5 (1.5-1.5)	18,955 (18,455-19,456)	3.2 (3.2-3.3)
Moderate active	29,958 (28,978-30,937)	5.2 (5.0-5.4)	1.5 (1.5-1.5)	19,159 (18,639-19,678)	3.2 (3.1-3.3)
High Active	26,321 (25,396-27,247)	4.6 (4.4-4.7)	1.4 (1.4-1.4)	17,478 (16,988-17,969)	2.9 (2.8-3.0)

Abbreviation: CI, confidence interval.

a Fitness activity status definitions: 1) not registered — not registered in the Vitality health promotion program; 2) inactive — earned points equivalent to 3 or fewer gym visits per year; 3) low active — earned points equivalent to 4 to 23 gym visits per year; 4) moderate active — earned points equivalent to between 24 to 48 gym visits per year; and 5) high active — earned points equivalent to more than 48 gym visits per year.

b South Africa rand, R1 = US $0.12.

c *P* < .001 for each activity group compared with the high active group (Tukey-Kramer *t* test for multiple comparisons).

**Table 4:** Hospital Admissions and Claims Experience of All Members, by Fitness Activity Status, Discovery Health, South Africa, 2006

**Fitness Activity Status** a	Mean Cost per Member, R^b^ (95% CI)^c^	Mean No. of Admissions per Member (95% CI)^c^	Mean Length of Stay per Member, d (95% CI)^c^
Not registered	8,644 (8,545-8,743)	0.42 (0.42-0.42)	1.7 (1.7-1.7)
Inactive	9,075 (8,966-9,184)	0.44 (0.44-0.44)	1.7 (1.7-1.7)
Low active	8,770 (8,560-8,980)	0.42 (0.42-0.43)	1.6 (1.6-1.6)
Moderate active	8,642 (8,428-8,856)	0.41 (0.40-0.42)	1.5 (1.5-1.6)
High active	7,540 (7,354-7,727)	0.36 (0.36-0.37)	1.4 (1.3-1.4)

Abbreviation: CI, confidence interval.

a Fitness activity status definitions: 1) not registered — not registered in the Vitality health promotion program; 2) inactive — earned points equivalent to 3 or fewer gym visits per year; 3) low active — earned points equivalent to 4 to 23 gym visits per year; 4) moderate active — earned points equivalent to between 24 to 48 gym visits per year; and 5) high active — earned points equivalent to more than 48 gym visits per year.

b South Africa rand, R1 = US $0.12.

c *P* < .001 for each activity group compared with the high active group (Tukey-Kramer *t* test for multiple comparisons).

**Table 5:** Difference in Hospital Admission Rates for the High Active Fitness Activity Group Compared With All Other Groups^a^, by Disease-Related Group, Discovery Health, South Africa, 2006

**Disease-Related Group**	% Difference^b^
Cardiovascular	−8
Digestive	−12
Nervous and musculoskeletal system	−16
Cancer	−35
Kidney and urinary tract	−20
Respiratory	−19
Mental	−35
Endocrine, nutritional, and metabolic	−20
Overall	−16

a Fitness activity status definitions: 1) not registered — not registered in the Vitality health promotion program; 2) inactive — earned points equivalent to 3 or fewer gym visits per year; 3) low active — earned points equivalent to 4 to 23 gym visits per year; 4) moderate active — earned points equivalent to between 24 to 48 gym visits per year; and 5) high active — earned points equivalent to more than 48 gym visits per year.

b *P* < .001 vs high active for all disease-related groups (Tukey-Kramer *t* test for multiple comparisons).
